# Gastrointestinal toxicity of vorinostat: reanalysis of phase 1 study results with emphasis on dose-volume effects of pelvic radiotherapy

**DOI:** 10.1186/1748-717X-6-33

**Published:** 2011-04-08

**Authors:** Åse Bratland, Svein Dueland, Donal Hollywood, Kjersti Flatmark, Anne H Ree

**Affiliations:** 1 Department of Oncology, Norwegian Radium Hospital - Oslo University Hospital, Oslo, Norway; 2 Department of Gastroenterological Surgery, Norwegian Radium Hospital - Oslo University Hospital, Oslo, Norway; 3 Department of Tumor Biology - Institute for Cancer Research, Norwegian Radium Hospital - Oslo University Hospital, Oslo, Norway; 4 Academic Unit of Clinical and Molecular Oncology, Institute of Molecular Medicine, Trinity College Dublin, Dublin, Ireland; 5 Department of Oncology, Akershus University Hospital, Lørenskog, Norway; 6 Institute of Clinical Medicine, University of Oslo, Oslo, Norway

## Abstract

**Background:**

In early-phase studies with targeted therapeutics and radiotherapy, it may be difficult to decide whether an adverse event should be considered a dose-limiting toxicity (DLT) of the investigational systemic agent, as acute normal tissue toxicity is frequently encountered with radiation alone. We have reanalyzed the toxicity data from a recently conducted phase 1 study on vorinostat, a histone deacetylase inhibitor, in combination with pelvic palliative radiotherapy, with emphasis on the dose distribution within the irradiated bowel volume to the development of DLT.

**Findings:**

Of 14 eligible patients, three individuals experienced Common Terminology Criteria of Adverse Events grade 3 gastrointestinal and related toxicities, representing a toxicity profile vorinostat has in common with radiotherapy to pelvic target volumes. For each study patient, the relative volumes of small bowel receiving radiation doses between 6 Gy and 30 Gy at 6-Gy intervals (V6-V30) were determined from the treatment-planning computed tomography scans. The single patient that experienced a DLT at the second highest dose level of vorinostat, which was determined as the maximum-tolerated dose, had V6-V30 dose-volume estimates that were considerably higher than any other study patient. This patient may have experienced an adverse radiation dose-volume effect rather than a toxic effect of the investigational drug.

**Conclusions:**

When reporting early-phase trial results on the tolerability of a systemic targeted therapeutic used as potential radiosensitizing agent, radiation dose-volume effects should be quantified to enable full interpretation of the study toxicity profile.

**Trial registration:**

ClinicalTrials.gov: NCT00455351

## Findings

### Context

With current advances in molecular radiobiology, strategies for improving efficacy of clinical radiotherapy are increasingly focused on investigating targeted compounds as radiosensitizing agents. The accepted investigational sequence for clinical evaluation consists of initial toxicity assessment of the systemic compound in combination with radiation, and the conventional 3+3 expansion cohort design remains the prevailing method for conducting phase 1 trials in cancer therapy [1]. In radiotherapy, the location of the disease predetermines the potential normal tissues that will be exposed. Unless the study design mandates that patients' disease sites are restricted to specific anatomic sites, the 3+3 expansion cohort model may be unsuitable for assessing the rate of adverse events and overall normal tissue toxicity as study endpoints.

Furthermore, in radiotherapy, toxic complications are both common and acceptable, and adverse events are often interrelated. Radiation-induced early toxicity is commonly experienced as a transient phenomenon either during the therapy course or within a few weeks of treatment completion, typically in normal tissues with a hierarchical proliferative structure, such as the mucosal lining of the gastrointestinal tract [2]. When combining radiation with targeted therapeutics that have the potential to modulate radiation-induced cellular responses, additive or synergistic normal tissue effects should be anticipated.

It is widely recognized that irradiation of large volumes is associated with a heightened risk of normal tissue toxicity. For example, in protocols applying irradiation of the bowel with two- to four-field techniques, moderate to severe acute gastrointestinal toxicity, primarily diarrhea, is observed in a significant fraction of patients. Furthermore, the probability and severity of such effects increase with the size of the therapeutic target volume and the dose per fraction [3]. Recently, attempts to quantify dose-volume effects within the small bowel have been reported, and data suggests that radiation-induced acute small bowel toxicity can be predicted by threshold estimates for varying dose-volume combinations [4].

Consequently, studies that are designed as early investigations into the safety of combining targeted therapeutics with pelvic radiotherapy may be particularly challenging to conduct as acute bowel toxicity is frequently encountered with radiation alone. In this setting, it may be difficult to decide whether or not a toxic event occurring during treatment is greater than might be expected for either of the therapeutic components and specifically whether the event should be considered a dose-limiting toxicity (DLT) of the systemic agent.

We have recently conducted a phase 1 study, PRAVO - Pelvic Radiation and Vorinostat, on vorinostat (Merck & Co., Inc., Whitehouse Station, NJ, USA), a histone deacetylase inhibitor, in combination with pelvic palliative radiotherapy for advanced gastrointestinal carcinoma [5], and have experienced methodological limitations in determining maximum-tolerated dose (MTD) and DLT. Hence, in the current report, we have reanalyzed the study toxicity data with emphasis on the relevance of the dose distribution within the irradiated bowel volume to the development of DLT. All DLTs reported by the study patients were gastrointestinal and related adverse events, representing a toxicity profile vorinostat has in common with radiotherapy to pelvic target volumes [6].

## Methods

The PRAVO study was approved by the Regional Committee for Medical and Health Research Ethics and was performed in accordance with the Declaration of Helsinki. Written informed consent was required for participation.

The study objective was to determine tolerability of vorinostat, defined by DLT and MTD, when administered concomitantly with palliative radiation to pelvic target volumes. The principal eligibility criterion was pelvic carcinoma scheduled to receive palliative radiation to 30 Gy in 3-Gy daily fractions. Other details on eligibility are given in the initial report [5]. The radiotherapy was delivered to target volumes (macroscopic tumor burden, as depicted by magnetic resonance imaging, with appropriate margins) determined by computed tomography (CT)-based conformal planning. Median values for minimum and maximum doses to the internal target volume were 28.3 Gy (range 26.1-28.9 Gy) and 31.4 Gy (range 30.8-33.6 Gy), respectively. The study adopted the standard 3+3 expansion cohort design, where patients were enrolled onto sequential dose levels of vorinostat, as previously detailed [5].

Toxicity was recorded continuously during treatment and was reexamined two and six weeks after treatment completion, and was graded according to Common Terminology Criteria for Adverse Events version 3.0. DLT was defined as grade ≥3 toxicity. A treatment delay longer than one week due to toxicity was also considered a DLT.

Fourteen of the 16 study patients had treatment-planning CT scans visualizing the entire abdominal and pelvic cavities and were evaluable for this reanalysis. Individual loops of small bowel were contoured on each slice of the planning CT scans, enabling the generation of total small bowel dose-volume histograms [4]. For each of the 14 patients, the relative volumes of small bowel receiving radiation doses of 6 Gy to 30 Gy, defined as V6-V30, were recorded at 6-Gy intervals.

The data reported here is solely descriptive, and no statistical adaptation has been undertaken.

## Results

Patient baseline characteristics and the complete data on adverse events have been described previously [5]. Of note, the locations of the radiotherapy target lesions were heterogeneous within the pelvic cavity or surrounding anatomic structures, and several patients had multiple targets. Fourteen patients were eligible for reanalysis of the toxicity data with regard to radiation dose-volume profiles (Table 1).

**Table 1:** The individual patients' radiation dose-volume relationships, vorinostat dose, and any grade 3 adverse event

Age (years)	Gender	GTV (ccm)	ITV (ccm)	SBV (ccm)	V6 (%)	V12 (%)	V18 (%)	V24 (%)	V30 (%)	Vorinostat dose (mg)	DLT grade 3 adverse event	Other grade 3 adverse event
87	female	285	648	823.8	79	74	70	67	40	300	anorexia, fatigue	
81	female	72.2	380	990.9	63	41	22	18	0	300		
66	female	171	483	2292	45	37	19	15	3	200		rash (following cetuximab)
49	female	89.5	323	1291	43	38	33	25	11	200		
47	female	198	414	2440	42	37	34	30	14	300		
83	female	197	549	1114	41	29	24	19	3	400	diarrhea, anorexia, hyponatremia	
55	male	87.7	867	1811	34	23	18	16	6	400		
75	female	36.7	277	1516	31	14	11	8	0	400	diarrhea, fatigue, hypokalemia	
62	male	114	324	2180	19	5	3	2	0	400		
77	male	153	650	1972	18	7	3	3	1	300		fatigue (during pneumonia)
45	female	58.1	175	2163	16	7	5	4	0	400		
82	male	75.5	330	2946	15	4	1	1	0	300		fatigue (during pneumonia)
77	female	164	625	1901	5	2	1	0	0	100		
85	female	60.1	180	1256	4	2	2	1	0	400		

Abbreviations: GTV = gross tumor volume; ccm = cubic centimeter; ITV = internal target volume; SBV = small bowel volume; V6-V30 = the relative volumes of small bowel receiving radiation doses of 6-30 Gy; DLT = dose-limiting toxicity.

Six of the 14 patients experienced grade 3 adverse events; however, in three patients, the reported event was considered to be unrelated to the study treatment: one in a patient receiving 200 mg vorinostat and reporting a grade 3 acneiform rash following commencement of cetuximab, and two in patients at 300 mg vorinostat with pneumonia, who reported grade 3 fatigue that rapidly resolved on antibiotic treatment.

The remaining three cases of grade 3 adverse events were considered to be treatment-related and were therefore documented as true DLTs. One of six patients receiving 300 mg vorinostat reported grade 3 anorexia and fatigue. At 400 mg vorinostat, two of six patients reported grade 3 diarrhea, with one patient developing synchronous grade 3 anorexia and hyponatremia and the other experiencing grade 3 fatigue and hypokalemia. Since one of six patients at 300 mg vorinostat and two of six patients within the 400 mg dose cohort reported a DLT, the MTD of vorinostat, according to conventional phase 1 study design, was determined to be 300 mg once daily.

For each of the 14 patients, data on absolute volumes of gross tumor, internal radiation target, and total small bowel, relative volumes of small bowel receiving radiation doses between 6 Gy and 30 Gy at 6-Gy intervals (V6-V30), and the daily vorinostat dose is summarized in Table 1. Within the table, patients are listed in descending order with reference to the V6 values. Of particular note, the single patient that experienced a DLT in the vorinostat 300 mg dose cohort had the greatest V6, and all her additional dose-volume estimates (V12-V30) were considerably higher than in any other patient assessed.

In the vorinostat 400 mg dose cohort, however, the radiation dose-volume records for the two patients reporting DLTs ranked towards the middle of the tabulated list. In these two patients, the relative volumes of irradiated small bowel across all radiation doses (V6-V30) appeared to be within the same order of magnitude and ranked first and third within the vorinostat 400 mg dose cohort separately. Three of the remaining four patients in this dose cohort had considerably lower values of V6-V30.

Because some patients had radiotherapy target lesions located in anatomic structures outside the pelvic cavity, such as the perineum or pelvic wall, their irradiated small bowel volumes were smaller than the internal radiation target volumes.

## Implications

As typically may be the case with phase 1 studies, the size of the PRAVO study population was small and few adverse events were recorded. Thus, the resulting data is descriptive and not subject for expedient handling statistically. Nevertheless, following this reanalysis of the PRAVO toxicity data, it seems probable that the single patient reporting a DLT at the vorinostat 300 mg dose level may have experienced an adverse radiation dose-volume effect rather than a toxic effect of the investigational drug. In the remaining four patients within the 300 mg dose cohort, and in all other study patients reported here, the relative volumes of small bowel receiving radiation doses of 12-30 Gy (V12-V30) were substantially smaller. However, our previous conclusion that vorinostat 300 mg once daily defines the MTD in this therapeutic setting [5] holds true, since the two patients (of six) reporting DLTs at 400 mg vorinostat had radiation dose-volume records (V6-V30) that essentially were indistinguishable from the estimates in patients without any treatment-related grade 3 adverse events. These observations suggest that, when applying an early-phase study design to evaluate tolerability of a systemic targeted therapeutic combined with radiotherapy, the contribution of radiation dose-volume effects to the observed toxicity should be quantified and reported in a standardized manner to enable full interpretation of the study toxicity profile.

When applying the standard 3+3 expansion cohort design to assess relevant normal tissue toxicities in radiotherapy trials, we propose that the potential disease site being irradiated should be clearly specified as study eligibility criterion. Unlike early-phase studies with systemic therapies, where location of disease manifestations presumably is less critical for evaluation of treatment tolerability, the anatomic site of the target lesions determines the normal organs exposed in radiotherapy.

The PRAVO study was designed as an initial investigation examining the safety of a histone deacetylase inhibitor employed as radiosensitizing component of pelvic radiotherapy. Importantly, within the study design, small bowel toxicity was an anticipated outcome parameter, since single-agent vorinostat is known to be tolerated at 400 mg daily for continuous dosing, with the most common side effects being fatigue and gastrointestinal toxicities [6]. Consequently, the toxicity profiles of pelvic radiation and vorinostat might overlap or potentially be synergistic. We suggest that in a phase 1 trial setting, where overlapping toxicities between a targeted systemic compound and radiation are anticipated, it would be highly beneficial if detailed radiation dose-volume constraints are described within the treatment protocol.

The pragmatic 3+3 expansion cohort design has been the prevailing method of documenting adverse events associated with administration of new drugs, as it requires no modeling of the dose-toxicity curve beyond the classical assumption for cytotoxic agents, including radiotherapy, that toxicity increases with dose [1]. In the context of combining a systemic targeted agent with radiotherapy, it is acknowledged that the delivered radiation dose may on occasion be close to or even at the limits of normal tissue tolerance. The awareness of this possibility is a strong argument in favor of precise dose escalation methods for the systemic agent and/or radiation schedule, that are simple and convenient to administer and that equally take account of potential radiation dose-volume effects.

Learning from this reanalysis of the PRAVO study outcome data, albeit derived from few reported adverse events in a small study population, radiation dose-volume effects should be quantified when reporting early-phase trial results on the tolerability of a systemic targeted therapeutic used as potential radiosensitizing agent. We believe there are methodological requirements in future early-phase trials utilizing novel radiosensitizers, particularly with regard to patient eligibility criteria, predetermining specific tumor sites and, as a consequence, the radiotherapy target volume.

## Competing interests

The authors declare that they have no competing interests.

## Authors' contributions

ÅB contributed to the design of the study and generated the dose-volume data. SD managed the provision of the clinical information. DH contributed to the concept and design of the study. KF managed the patient database. AHR contributed to the concept and design of the study, managed the patient database, and drafted the final manuscript. All authors contributed to data analysis and interpretation, and read and approved the final version of the manuscript.
